# Counting co-occurring diseases to predict mortality is as accurate as multimorbidity indices: an external validation study

**DOI:** 10.1093/ageing/afag150

**Published:** 2026-06-05

**Authors:** Premysl Velek, Marije J Splinter, Lucy Stirland, Annemarie Luik, Patrick J Bindels, Bruno Stricker, Evelien I T de Schepper, Barbara C van Munster, Rikje Ruiter, Guy Brusselle, Maryam Kavousi, Silvan Licher

**Affiliations:** Department of Epidemiology, Erasmus University Medical Center Rotterdam, Rotterdam, The Netherlands; Department of General Practice, Erasmus University Medical Center Rotterdam, Rotterdam, The Netherlands; Department of Epidemiology, Erasmus University Medical Center Rotterdam, Rotterdam, The Netherlands; Division of Psychiatry, The University of Edinburgh Centre for Clinical Brain Sciences, Edinburgh, UK; Department of Epidemiology, Erasmus University Medical Center Rotterdam, Rotterdam, The Netherlands; Department of General Practice, Erasmus University Medical Center Rotterdam, Rotterdam, The Netherlands; Department of Epidemiology, Erasmus University Medical Center Rotterdam, Rotterdam, The Netherlands; Department of General Practice, Erasmus University Medical Center Rotterdam, Rotterdam, The Netherlands; University Medical Center Groningen, University Center of Geriatric Medicine, University of Groningen, Groningen, The Netherlands; Department of Epidemiology, Erasmus University Medical Center Rotterdam, Rotterdam, The Netherlands; Internal Medicine, Maasstad Hospital, Rotterdam, Zuid-Holland, The Netherlands; Department of Epidemiology, Erasmus University Medical Center Rotterdam, Rotterdam, The Netherlands; Respiratory Medicine, University Hospital Ghent, Gent, Oost-Vlaanderen, Belgium; Respiratory Medicine, Erasmus Medical Center, Rotterdam, Zuid-Holland, The Netherlands; Department of Epidemiology, Erasmus University Medical Center Rotterdam, Rotterdam, The Netherlands; Department of Epidemiology, Erasmus University Medical Center Rotterdam, Rotterdam, The Netherlands; Department of General Practice, Erasmus University Medical Center Rotterdam, Rotterdam, The Netherlands

**Keywords:** multimorbidity, non-communicable diseases, cohort study, external validation, older people

## Abstract

**Background:**

A systematic review recommended seven multimorbidity indices for predicting mortality. However, their performance has not been assessed in a head-to-head comparison. We externally validated these indices and determined their performance compared to counting co-occurring diseases.

**Setting:**

Within the prospective Rotterdam Study in the Netherlands, we constructed seven specific sub-cohorts, selected from 14 926 community-dwelling older adults to match the target population of the selected multimorbidity indices.

**Methods:**

We calculated prediction scores according to the indices’ original methods and used these as predictors in logistic regression models with all-cause mortality as outcome. We assessed their performance and compared it to four benchmark models fitted on the same index-specific samples. These models were based on (i) age and sex; (ii) counts of co-occurring diseases, age and sex; (iii) counts of co-occurring diseases associated with mortality, age and sex; and (iv) individual diseases as separate predictors, age and sex.

**Results:**

The total population sizes of the seven sub-cohorts ranged from 2409 to 9045 participants. The mean age of the populations ranged from 59.4 to 77.0 years; the proportion of women ranged from 56.0% to 61.8% (excluding single-sex indices). The absolute risk for mortality ranged from 0.9% to 13%. Discriminative performance of the indices and corresponding count models was nearly identical across all indices (maximum difference in C-statistic: 0.06), yet higher than age-and-sex models. Absolute accuracy of the prediction scores was similar across all models (maximum improvement in Brier score: 4%). Calibration was poor in four out of seven indices, all of which had a follow-up time of 2 years or less.

**Conclusion:**

Counting co-occurring diseases is as accurate in predicting all-cause mortality in the general population as using multimorbidity indices. These findings imply that counting diseases is the more practical and reliable way of providing prognosis to patients with multimorbidity in a population of community-dwelling adults.

## Key Points

- Counting co-occurring diseases is as accurate as complex multimorbidity indices.
- Counting co-occurring diseases is recommended as the more practical way of assessing multimorbidity in older adults.
- There are seven recommended multimorbidity indices that aim to predict the risk of all-cause mortality among older adults.
- Yet, it is unclear whether these indices have added value compared to the simple count of co-occurring diseases, or age and sex.

## Introduction

Multimorbidity is one of the main challenges facing global healthcare systems. It is typically defined as the co-occurrence of two or more chronic diseases, which provides an intuitive summary of both burden of disease and burden of treatment [1]. While the approach of counting diseases is one of the most commonly used and recommended methods to quantify multimorbidity [1], it may not fully reflect the potential unequal effects of co-occurring diseases on health outcomes [2]. To address these potential limitations, various indices have been developed in an attempt to capture the complexity of multimorbidity through a single metric. These indices assign weights to different components, such as disease type, disease severity, dispensed drugs or lifestyle factors, based on their association with the outcome under study. The weights are then summed to generate a score for each individual patient, reflecting the estimated risk of the outcome.

There are various multimorbidity indices that have been used in many different contexts: from selection criteria for healthcare interventions, through clinical decision-making and risk assessment, to tools to adjust for potential confounding [2, 3]. A systematic review identified 35 indices focused on community-dwelling older adults and recommended 12 for clinical and research use, based on their design, risk of bias and published data on performance [2]. Their predicted outcomes are diverse, yet seven focus on mortality, given its relevance for both clinical practice and research. Several of these seven indices have been externally validated or benchmarked against widely used indices, such as the Charlson Comorbidity Index [4]. However, it is not clear whether these indices have added predictive value beyond simpler measures such as counting diseases, or only age and sex.

In this study, we evaluated multimorbidity indices that predict all-cause mortality among community-dwelling populations, using long-term follow-up data from the Rotterdam Study. Importantly, we assessed whether these indices provide added value compared to simpler models based on disease counts, and age and sex alone in a head-to-head comparison.

## Methods

### Selection of multimorbidity indices

Seven out of 12 indices recommended by a systematic review of multimorbidity indices were included in our analyses [2]. We refer to these indices by the name of the first author and the year of publication: (i) Von Korff, 1992 [5]; (ii) Desai, 2002 [6]; (iii) Fan, 2002 [7]; (iv) Lee, 2006 [8]; (v) Tooth, 2008 [9]; (vi) Quan, 2011 (updated Charlson Comorbidity Index) [10]; and (vii) Robusto, 2016 [11].

Five of these seven indices assigned different weights to a set of diseases (Desai, 2002; Fan, 2002; Lee, 2006; and Quan, 2011) and calculated the individual prediction score by summing up the weights corresponding to diseases co-occurring within an individual. Two indices (Von Korff, 1992 and Robusto, 2016) relied on weights assigned to drug prescriptions rather than disease. All seven indices had community-dwelling adults as their target population [2]. Two indices (Desai, 2002 and Quan, 2011) used a population of previously hospitalised older adults and assessed all-cause mortality following a hospital discharge. Detailed information about each multimorbidity index is presented in Appendix 1a and 1b.

### Study population for external validation

We used data from the population-based Rotterdam Study, a prospective cohort study aimed at investigating the aetiology and natural history of chronic diseases in mid- and late-life [12]. The Rotterdam Study was initiated in 1990 and consisted of 7983 residents of the district Ommoord in Rotterdam, the Netherlands, who were 55 years and older (‘RS-I’). The cohort was expanded with additional study waves in 2000 (*N* = 3011, ‘RS-II’), 2006 (*N* = 3932, minimum age 45 years, ‘RS-III’) and 2016 (*N* = 3005, minimum age 40 years, ‘RS-IV’). As follow-up of RS-IV was not yet complete, we only used data from the first three cohorts, comprising 14 926 participants in total. To replicate the original indices as closely as possible, we used specific sub-samples of the Rotterdam Study that aligned with the target populations. This included accounting for variations in the types and number of diseases considered, as well as the age and sex of participants, and total follow-up duration. We excluded participants with missing data for variables required to calculate the prediction score. This study is reported according to the Transparent Reporting of a Multivariable Prediction Model for Individual Prognosis or Diagnosis (TRIPOD) Statement [13].

### Ascertainment of disease status and collection of lifestyle data

Participants visit the research centre of the Rotterdam Study upon study entry and during subsequent follow-up every 3 to 6 years for repeated examinations. Additional information is obtained through linkage with medical records for general practitioners and medical specialists, with the Dutch national cancer registry and public pathology database. These records are verified by research assistants to assess the incidence of any of the chronic diseases of interest. Final diagnoses are made during consensus meetings, which consist of research physicians and medical specialists. Information about drug use was obtained through linkage with pharmacies in which the participants were registered. Lifestyle information was collected through interviews [12]. A summary of data collection methods is available in Appendix 2.

### Assessment of all-cause mortality

We determined the date of death by notification by the municipal administration, general practitioner (GP) or nursing home, or by the family of the deceased. Living status was assessed by the most recent date of a home interview or research visit for the Rotterdam Study, or the last date of checking GP files, whichever came first.

### Statistical analyses

First, we determined the baseline prevalence of all diseases and other variables included in each index. Given the variation in data collection methods and disease definitions across the indices, we applied the definitions that were used within the Rotterdam Study. Cancer (sub-)types were classified according to ICD-9 or ICD-10 codes, drug prescriptions were defined using ATC-codes.

When diagnoses in the original index were self-reported, we identified the closest equivalent within the Rotterdam Study by selecting a single disease, a subset of diseases or a group of diseases that best matched the original definition. Further information about mapping is available in Appendix 3a.

Second, we calculated the respective prediction scores for each participant according to each index. Each prevalent disease and, if relevant, other variables at baseline were assigned an integer value (weight), as reported by the index authors. Following identical methods as in the original papers, the sum of these weights determined the prediction score. The weights assigned to individual diseases and drug prescriptions are available in Appendix 3a and b.

Third, we fitted five regression models to each sample population with all-cause mortality as outcome. We used logistic regression in all models to match methods from original papers and to maintain consistency in performance evaluation across indices. In the first model, the ‘index model,’ we included the prediction score as described above and any other variable which was included in the original papers (typically age and/or sex). We developed four comparator models with different predictors, against which we evaluated the index model: (i) the ‘base model’ with only age at baseline and sex, which was included as a comparator model to assess whether more complex models provide added value in predicting all-cause mortality; (ii) the ‘count model’ containing age and sex, and a simple count of co-occurring diseases. We considered the following 10 chronic diseases: asthma, cancer (all types excluding non-melanoma skin cancer), chronic obstructive pulmonary disease (COPD), coronary heart disease, type 2 diabetes, dementia, depression, heart failure, Parkinsonism and stroke. The selected diseases have high prevalence and burden of disease in older populations in developed countries and are all in the core list of diseases recommended for multimorbidity studies [14]. (iii) The ‘count mortality model’ containing age and sex and counts of co-occurring diseases with high prevalence and high risk of mortality: coronary heart disease, stroke, dementia, chronic kidney disease, COPD, lung cancer, colorectal cancer and major depressive disorder [15]. (iv) The ‘exact mortality model,’ includes the same set of diseases as the ‘count mortality model’ but each individual disease is included in the model as a separate variable.

The four comparator models were then applied to the seven sample populations constructed to match the target population of the seven multimorbidity indices. As all models were applied to the same sample population for each separate index, the four comparator models served as benchmarks against which we compared the performance of each index model. This ensured a fair evaluation of the indices.

To evaluate the predictive performance of all models, we used the C-statistic, Brier Skill Score, Net Reclassification Improvement (NRI) and Integrated Discrimination Index (IDI). A description and interpretation of the evaluation metrics are available in Appendix 4, and formulas for calculating the metrics are presented in Appendix 5. All metrics were corrected for optimism through a bootstrapping procedure, using 1500 bootstrap samples with replacement. We computed corresponding 95% confidence intervals using the location-shifted bootstrap method [16]. Whenever possible, we also assessed calibration using calibration plots, which show the agreement between predicted and observed probabilities of mortality [17]. Data were handled and analysed using R version 4.2.1.

### Sensitivity analyses

Three indices were developed for populations of a specific age (Tooth, 2008 and Desai, 2002: 70 years and older) or sex (Tooth, 2008 and Fan, 2002). To assess generalisability to other groups, we applied these indices to a sample without any age or sex restrictions and compared them to the count and base models fitted to the same data.

Two indices (Desai, 2002 and Quan, 2011) only considered diabetes with end-organ damage or diabetes with chronic complications. As data on diabetes severity were not available within the Rotterdam Study, we calculated the upper bound of the indices’ performance by considering the hypothetical scenario in which diabetes with end-organ damage or chronic complications always led to mortality. In this case, participants with diabetes who died during follow-up kept their diagnoses of diabetes, while participants with diabetes who did not die were considered as having diabetes without end-organ damage or chronic complications.

### Role of the funding source

The Rotterdam Study is funded by Erasmus MC University Medical Center and Erasmus University Rotterdam; Netherlands Organization for Health Research and Development (ZonMw); the European Commission; and the Municipality of Rotterdam. This study was supported by ZonMw as part of the SHIELD-U2 project (grant number 10430362220003) and VENI-project (grant number 09150162410227). The funders had no role in study design, data collection and analysis, decision to publish or preparation of the manuscript.

### Institutional review board approval

The Rotterdam Study has been approved by the Medical Ethics Committee of the Erasmus MC (registration number MEC 02.1015) and by the Dutch Ministry of Health, Welfare and Sport (Population Screening Act WBO, licence number 1071272-159521-PG). Written informed consent was obtained from all participants.

## Results

### Population characteristics

The total population sizes ranged from 2409 to 9045 participants. Indices with older target populations (Tooth, 2008 and Desai, 2002) and single-sex indices (Fan, 2002 and Tooth, 2008) had the smallest population sizes (Table 1). The mean age of the populations ranged from 59.4 (sample for Von Korff, 1992) to 77.0 (sample for Desai, 2002) years. Five indices were developed for both women and men. The proportion of women in samples for these indices ranged from 56.0% (Von Korff, 1992) to 61.8% (Desai, 2002), with older populations containing more women. The absolute risk for mortality ranged from 0.9% (Quan, 2011; 1-year follow-up) to 13% (Tooth, 2002; 6-year follow-up).

**Table 1:** Baseline characteristics of the external validation sample populations selected from the Rotterdam Study.

	Rotterdam Study (*N* = 14 926)	Robusto, 2016 (*N* = 8442)	Quan, 2011 (*N* = 7531)	Tooth, 2008 (*N* = 2350)	Lee, 2006 (*N* = 3597)	Desai, 2002 (*N* = 2637)	Fan, 2002 (*N* = 1925)	Von Korff, 1992 (*N* = 8501)
**Mean age, years (SD)**	65.9 (10.5)^a^	64.7 (9.5)	68.9 (9.1)	73.6 (6.6)	69.0 (7.4)	77.0 (6.3)	71.7 (7.2)	64.6 (9.5)
**Women (%)**	8823 (59.1)	4802 (56.8)	4343 (58.0)	2350 (100.0)	2078 (57.7)	1610 (61.0)	0 (0)	4832 (56.8)
**Follow-up time**	n.a.	6 years	1 year	6 years	4 years	1 year	2 years	1 year
**Died during follow-up (%)**	n.a.	895 (11.0)	110 (1.0)	296 (13.0)	108 (3.0)	82 (3.0)	69 (4.0)	50 (1.0)
**Northwest European ethnic background** ^**b**^ ^**,**^ ^**c**^ **(%)**	12 595 (84.4)	7676 (90.9)	6806 (90.4)	2113 (89.9)	3308 (92.0)	2349 (89.1)	1755 (91.2)	7730 (90.9)
**Educational attainment** ^**d**^ **(%)**								
**Primary education**	2712 (18.2)	982 (11.6)	842 (11.2)	490 (20.9)	393 (10.9)	392 (14.9)	151 (7.8)	988 (11.6)
**Low/intermediate general or lower vocational**	5770 (38.7)	3435 (40.7)	3091 (41.0)	1192 (50.7)	1615 (45.0)	1150 (43.6)	579 (30.1)	3457 (40.7)
**Intermediate vocational or higher general**	2139 (14.3)	1536 (18.2)	1376 (18.3)	118 (5.0)	490 (13.6)	280 (10.6)	421 (21.9)	1551 (18.2)
**Higher vocational or university**	3878 (26.0)	2479 (29.4)	2189 (29.1)	550 (23.4)	1099 (30.6)	815 (30.9)	762 (39.6)	2495 (29.3)

*N*, number of participants selected for the validation sample; CCI, Charlson Comorbidity Index; n.a., not applicable, sd, standard deviation.

^a^Age at entering the Rotterdam study.

^b^Classified according to the Australian Standard Classification of Cultural and Ethnic Groups (ASCCEG).

^c^Number of participants with missing data: Rotterdam Study: 1718 (12%), Robusto, 2016: 351 (4%); Quan, 2011: 350 (5%); Tooth, 2008: 191 (8%); Lee, 2006:199 (6%); Desai, 2002: 235 (9%); Fan, 2002: 116 (6%); Von Korff, 1992: 351 (4%).

^d^Number of participants with missing data were less than 1% for all sample populations except for the Rotterdam Study as a whole in which the number of missing data was 422 (2.8%).

### Discrimination, absolute accuracy and net reclassification improvement

**Figure 1:**
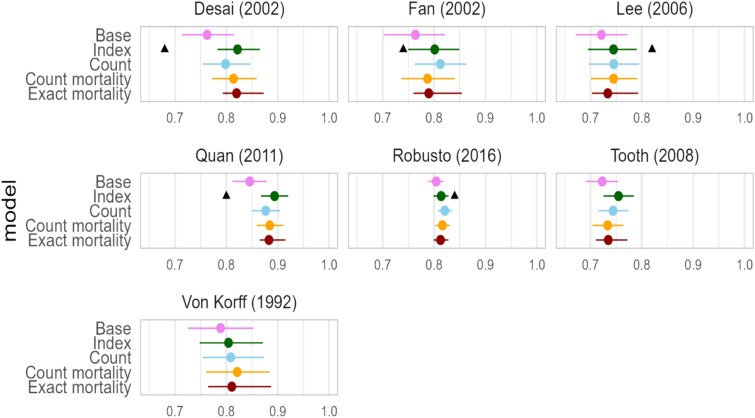
Discrimination of the ‘base,’ index, ‘count,’ ‘count mortality’ and ‘exact mortality’ models. If applicable, the C-statistic reported in the original article is visualised with a triangle (▲).

All performance values are presented in Table 2. A forest plot of the C-statistics across all models is visualised in Figure 1.

**Table 2:** Performance statistics of the index model in comparison to the ‘base’ model, two ‘count’ models and the ‘exact mortality’ model.

Sample for	Metric	Base	Index	Count	Count mortality	Exact mortality
**Tooth, 2008**	C-statistic	0.72 (0.69:0.75)	0.75 (0.73:0.78)	0.74 (0.72:0.77)	0.73 (0.70:0.76)	0.73 (0.71:0.77)
	BSS	0.100 (0.09:0.108)^a^	0.03 (0.01:0.05)	0.02 (0.01:0.04)	0.01 (0:0.02)	0.02 (0.01:0.05)
	NRI—events	–	−0.06 (−0.14:0.02)	0.23 (0.12:0.34)	−0.01 (−0.12:0.1)	−0.35 (−0.55:−0.03)
	NRI—non-events	*–*	0.42 (0.36:0.48)	0.19 (0.14:0.23)	0.47 (0.44:0.51)	0.70 (0.51:0.82)
	IDI	*–*	0.03 (0.01:0.05)	0.02 (0.01:0.04)	0.01 (0:0.02)	0.03 (0.01:0.05)
**Desai, 2002**	C-statistic	0.76 (0.71:0.81)	0.82 (0.78:0.87)	0.80 (0.75:0.85)	0.81 (0.77:0.86)	0.82 (0.79:0.87)
	BSS	0.029 (0.023:0.035)^a^	0.01 (0.00:0.05)	0.02 (0.01:0.06)	0.03 (0.01:0.07)	0.02 (0.01:0.09)
	NRI—events	*–*	0.15 (−0.01:0.32)	0.15 (−0.05:0.36)	0.23 (0.05:0.41)	0.38 (0.12:0.65)
	NRI—non-events	*–*	0.41 (0.30:0.50)	0.47 (0.34:0.51)	0.44 (0.30:0.60)	0.32 (0.25:0.46)
	IDI	–	0.03 (0.01:0.05)	0.03 (0.01:0.06)	0.04 (0.02:0.06)	0.06 (0.03:0.10)
**Lee, 2006**	C-statistic	0.72 (0.67:0.77)	0.74 (0.70:0.79)	0.75 (0.70:0.80)	0.74 (0.70:0.79)	0.73 (0.70:0.79)
	BSS	0.029 (0.024:0.034)^a^	0.01 (−0.01:0.03)	0.01 (0.00:0.03)	0.01 (−0.01:0.02)	0.00 (−0.01:0.03)
	NRI—events	*–*	−0.02 (−0.23:0.16)	0.06 (−0.1:0.21)	0.04 (−0.09:0.22)	0.06 (−0.18:0.25)
	NRI—non-events	*–*	0.11 (−0.10:0.31)	0.23 (−0.18:0.35)	0.03 (−0.21:0.47)	0.10 (−0.28:0.62)
	IDI	*–*	0.01 (0.00:0.03)	0.01 (0.00:0.03)	0.01 (0.00:0.02)	0.01 (0.00:0.03)
**Fan, 2002**	C-statistic	0.76 (0.7:0.82)	0.80 (0.75:0.85)	0.81 (0.76:0.86)	0.79 (0.74:0.84)	0.79 (0.76:0.85)
	BSS	0.033 (0.026:0.04)^a^	0.03 (0.00:0.08)	0.04 (0.01:0.10)	0.02 (0.00:0.06)	0.00 (0.00:0.09)
	NRI—events	*–*	0.17 (−0.03:0.38)	0.18 (−0.03:0.41)	0.14 (−0.04:0.31)	0.24 (−0.1:0.51)
	NRI—non-events	*–*	0.22 (0.00:0.34)	0.51 (0.32:0.63)	0.31 (0.24:0.41)	0.51 (0.00:0.68)
	IDI	–	0.03 (0.00:0.08)	0.05 (0.02:0.09)	0.02 (0.01:0.05)	0.05 (0.02:0.10)
**Quan, 2011**	C-statistic	0.85 (0.81:0.88)	0.89 (0.87:0.92)	0.88 (0.85:0.90)	0.88 (0.86:0.91)	0.88 (0.86:0.92)
	BSS	0.014 (0.011:0.016)^a^	0.02 (0.00:0.04)	0.02 (0.00:0.04)	0.01 (0.00:0.03)	0.01 (0:0.06)
	NRI—events	*–*	0.23 (0.08:0.36)	0.24 (0.06:0.4)	0.24 (0.08:0.4)	0.31 (0.09:0.48)
	NRI—non-events	*–*	0.46 (0.36:0.53)	0.39 (0.28:0.56)	0.46 (0.39:0.51)	0.27 (−0.01:0.49)
	IDI	*–*	0.03 (0.01:0.05)	0.02 (0.01:0.04)	0.02 (0.01:0.04)	0.04 (0.02:0.07)
**Robusto, 2016**	C-statistic	0.80 (0.79:0.82)	0.81 (0.80:0.83)	0.82 (0.81:0.84)	0.82 (0.80:0.83)	0.81 (0.80:0.83)
	BSS	0.079 (0.075:0.084)^a^	0.02 (0.01:0.03)	0.03 (0.02:0.05)	0.02 (0.01:0.03)	0.01 (0.00:0.03)
	NRI—events	*–*	−0.09 (−0.14:-0.03)	0.20 (0.13:0.26)	0.04 (−0.02:0.11)	0.01 (−0.07:0.1)
	NRI—non-events	*–*	0.46 (0.42:0.49)	0.28 (0.25:0.3)	0.44 (0.36:0.5)	−0.15 (−0.26:-0.04)
	IDI	–	0.02 (0.01:0.03)	0.03 (0.02:0.04)	0.02 (0.01:0.03)	0.02 (−0.02:0.03)
**Von Korff, 1992**	C-statistic	0.79 (0.72:0.85)	0.80 (0.75:0.87)	0.81 (0.75:0.87)	0.82 (0.76:0.88)	0.81 (0.76:0.89)
	BSS	0.006 (0.004:0.007)^a^	−0.01 (−0.01:0.01)	0.00 (0.00:0.02)	0.00 (−0.01:0.05)	−0.01 (−0.02:0.07)
	NRI—events	*–*	0.04 (−0.17:0.27)	0.05 (−0.19:0.3)	0.16 (−0.11:0.42)	0.14 (−0.09:0.38)
	NRI—non-events	*–*	0.23 (0.14:0.33)	0.30 (0.23:0.38)	0.34 (0.17:0.48)	0.03 (−0.17:0.42)
	IDI	–	0.00 (0:0.01)	0.01 (0:0.02)	0.02 (0.00:0.05)	0.03 (0.01:0.08)

BSS, Brier Skill Score (percent change in Brier score with respect to the base model).

NRI, Net Reclassification Improvement, the reference model is the ‘base model.’

NRI (events): proportion of events which the evaluated model assigned higher probability than the ‘base model.’

IDI: Integrated Discrimination Index—change in the discrimination slope (the difference between the mean probability of mortality among patients who died and patients who did not die).

^a^Reflects the Brier Score of the base model.

The index and the count models outperformed the age and sex only model (‘base model’) across the evaluated metrics. The performance of the index and the count models was similar as measured by the improvement in C-statistic, Brier Score and IDI as compared to the ‘base model.’ The increase in C-statistic ranged from 0.01 to 0.06. The ‘count model’ in Fan (2002) showed the largest improvement in the Brier Score (0.04). In the same sample, both the ‘count model’ and the ‘exact mortality model’ achieved the highest improvement in IDI values (0.05). The greatest improvements and the greatest variability in improvement across the models were observed for NRI, with the ‘count model’ within the Fan (2002) sample achieving the highest increase (0.51 NRI for non-events).

Several models performed worse than the ‘base model’ on one or more predictive metrics. For example, the index by Von Korff (1992) had poorer performance for the Brier Score, and the indices by Tooth (2008), Lee (2006) and Robusto (2016) performed worse in terms of NRI for events. Similarly, the ‘exact mortality model’ performed worse than the ‘base model’ in terms of the Brier Score in the Von Korff (1992) sample, the NRI for non-events in Robusto (2016) and the NRI for events in Tooth (2008). For the latter sample, the ‘count mortality model’ also showed a negative NRI.

### Calibration

**Figure 2:**
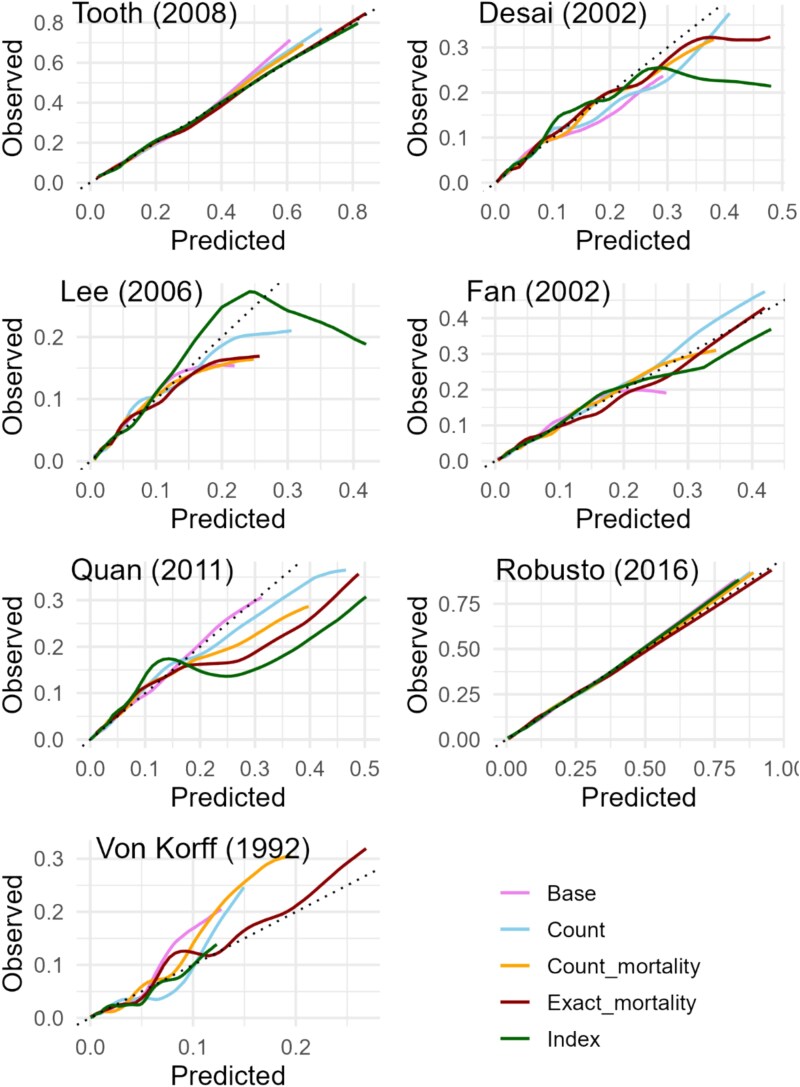
Calibration of the ‘base,’ index, ‘count,’ ‘count mortality’ and ‘exact mortality’ models.

The calibration plots for the index models of Desai (2002), Lee (2008) and Quan (2011), showed miscalibration (Figure 2). Calibration of the ‘count’ and ‘base’ models was better across most samples, though signs of miscalibration were observed in samples for Von Korff (1992) and Lee (2008). The ‘count mortality model’ was miscalibrated in the samples for Quan (2011) and Von Korff (1992), while the ‘exact mortality model’ was miscalibrated in the samples for Desai (2002), Lee (2008) and Quan (2011).

### Sensitivity analyses

The difference in discrimination between the index, ‘count’ and ‘base’ models decreased when indices with older people as their populations (Tooth, 2008, minimum age 72 years; Desai, 2002, minimum age 70 years) or with single-sex populations (Tooth, 2008 and Fan 2002) were applied to a population without any age and/or sex restrictions. The performance of the indices which included diabetes severity as a predictor (Desai, 2002 and Quan, 2011) improved when considering the hypothetical scenario in which diabetes with end-organ damage or chronic complications always led to mortality. However, the difference between the index and count models remained low. Complete results of the sensitivity analyses are in Appendix 6**.**

## Discussion

### Principal findings

In this external validation study, we showed that a count of co-occurring chronic diseases has comparable performance to more sophisticated multimorbidity indices in predicting all-cause mortality among community-dwelling middle-aged and older adults. Even though all multimorbidity indices had better relative discrimination ability (C-statistic) than the models solely based on age and sex, the same or very similar improvement was observed for the ‘count model’ and the other comparator models. This suggests that disease counts add similar information to the prediction models as multimorbidity indices.

However, the improvement observed in relative discrimination did not result in better absolute discrimination and more accurate estimates of all-cause mortality risk. Improvements in terms of the Brier Score and IDI were low or non-significant for all evaluated models. This indicates that the observed improvement would not be meaningful in clinical practice.

The miscalibration observed in three index models may be explained by differences between the original and our sample populations as those indices (Desai, 2002 and Quan, 2011) were developed for patients after hospital discharge, whereas the Rotterdam Study is based on community-dwelling participants [6, 10].

### Comparison with previous studies

There have been many studies assessing different versions of the Charlson Comorbidity Index and other comorbidity indices, yet these were often applied to hospitalised populations and did not use any benchmark model [18]. We have found six studies that compared weighted multimorbidity indices to disease counts. Five of them found no or minor differences in their discrimination performance as compared to counts [19–23]. The sixth study found that multimorbidity indices performed better than count models among older cancer patients. In this case, however, the weights were derived using the ACE-27 index developed specifically for this patient population [24]. Only one of these studies assessed absolute accuracy or calibration and found miscalibration in the weighted index [19].

### Strengths and limitations

We were able to evaluate the performance of recommended multimorbidity indices within a general population using extensive, long-term follow-up data, with accurate diagnoses ascertained by medical records, interviews and clinical assessments. We used a set of metrics, covering different aspects of model performance, to assess both relative and absolute discrimination, prediction accuracy, and calibration, and validated the models in populations as comparable as possible. There are also several limitations. First, we could not completely replicate the indices of Desai (2002), Fan (2002) and Quan (2011), as systematically collected data about pneumonia, acute renal failure, rheumatologic disease, liver disease and AIDS/HIV were not available to us. Similarly, our data did not include information about the severity of diabetes. We believe that including these data would not have led to a major increase in performance: these diseases either had low weights in the original studies and would not drastically change the overall score of individual patients (rheumatologic disease, liver disease and pneumonia), or their prevalence in the target population of Northwest European community-dwelling, non-hospitalised individuals is low (acute renal failure and HIV/AIDS). Our sensitivity analysis regarding diabetes severity only resulted in a modest improvement of the model performances. Another limitation is the limited generalisability of our findings to populations outside of the global West, given that the study populations of the original indices and the Rotterdam Study originate from this region. Finally, since this study is based on the most recent systematic review of multimorbidity indices, we did not include indices that were published after its search date [25–28].

### Implications for clinical practice and research

First, multimorbidity indices provide little added value when the objective is to generate absolute risk for a single patient. In our analyses, neither multimorbidity indices nor any of the comparator models meaningfully improved predictions beyond age and sex. Therefore, multimorbidity indices should not be interpreted as directly related to patients’ risk of mortality, as different multimorbidity score might translate to similar risks due to low accuracy and miscalibration.

Second, multimorbidity indices do offer additional information beyond age and sex in settings where relative accuracy is important, for instance in patient triage. However, counting the number of co-occurring diseases provides the same or similar insights, is more practical, and provides consistent results across different populations and age groups. Our findings show that such counts should also include diseases that are not highly predictive of mortality (e.g. mental health diseases), as these models performed better overall than models that only considered diseases with a high risk of mortality. Transparent reporting of diseases included in such counts would improve replicability [29].

Third, it can be argued that mortality alone may not be the most appropriate outcome against which to measure multimorbidity in a community setting. Focusing only on diseases highly associated with mortality leads to exclusion of many diseases with significant impact on quality of life, such as psychiatric diseases. None of the seven selected indices included any psychiatric disease or drugs for psychiatric disease. However, the coexistence of both physical and psychiatric diseases is common among older adults and the physical-psychiatric multimorbidity has been highlighted as one of the priorities for multimorbidity research [2]. Using multimorbidity indices as a general measure of multimorbidity may exclude patients who are not at high risk of premature mortality but who are nonetheless seriously affected by multimorbidity. Multimorbidity indices with unequal weights may lead to paradoxical situation whereby patients with a single disease highly associated with mortality have a higher multimorbidity score than patients with multiple diseases.

Finally, while we believe that multimorbidity is an important factor in managing older patients, complex multimorbidity indices do not account for its multifactorial nature. We believe that any successful measure of multimorbidity needs to consider both disease counts as well as diseases clustering, as different combination of diseases may have strong impact on health outcomes [30].

## Conclusions

In this population-based study, we showed that multimorbidity indices do not have higher predictive value for all-cause mortality in the general population as compared to the count of co-occurring diseases. These findings imply that counting diseases is the more practical way of measuring multimorbidity in a population of community-dwelling middle-aged or older adults. We encourage other researchers to perform additional external validation studies to test transportability and potential clinical utility of these indices to different settings.

## Supplementary Material

aa-25-3256-File002_afag150

## Research Data Transparency and Availability

Data from The Rotterdam Study can be made available to interested researchers upon request. Requests can be directed to the secretariat of the department of Epidemiology (secretariat.epi@erasmusmc.nl), or for more information visit http://www.ergo-onderzoek.nl/wp/contact. For legal and ethical restraints, and to ensure participant anonymity, we are unable to place data in a public repository.
