# Supplementary material for: Counting co-occurring diseases to predict mortality is as accurate as multimorbidity indices: an external validation study
Source: Age Ageing. 2026 Jun 5;55(6):afag150. doi: 10.1093/ageing/afag150 (PMC13240847; doi:10.1093/ageing/afag150)
Supplement: aa-25-3256-File002_afag150 [file aa-25-3256-file002_afag150.docx]

**Supplementary Data**

**Counting co-occurring diseases to predict mortality is as accurate as multimorbidity indices: an external validation study**

**Table of content**

| Appendix 1a: Descriptive information about the original multimorbidity indices | 3 |
| --- | --- |
| Appendix 1b: Characteristics of the indices | 4 |
| Appendix 2: Ascertainment of diseases and lifestyle information | 5 |
| Appendix 3: Disease mapping and weight assignment | 10 |
| Appendix 4. Performance evaluation methods  Appendix 5: Formulas for the performance metrics  Appendix 6: Results of the sensitivity analyses | 12  13  14 |
| References | 17 |

| **Appendix 1a \| Descriptive information about the original multimorbidity indices.** | | |
| --- | --- | --- |
| **Index** | | |
| Tooth, 2008  Country: Australia  Sample size: 5,217  Mean age: 75  % women: 100  Follow-up: 6 years | Included diseases | Heart disease, diabetes, stroke, cancer, low iron, Alzheimer’s disease, bronchitis/emphysema |
|  | Other variables | Age, sex |
|  | Data collection | Patient self-report |
|  | Weight calculation | Regression coefficients of Cox proportional hazards model |
| Desai, 2002  Country: United States  Sample size: 524  Mean age: 79  % women: 56  Follow-up: 1 year | Included diseases | CHF/cardiomyopathy, major stroke, pneumonia^a^, acute renal failure^a^, COPD/chronic lung disease, chronic renal failure, diabetes with end-organ damage^b^, cancer (solid tumour, localised), cancer (metastatic), cancer (lymphoma, leukaemia) |
|  | Other variables | Age, sex |
|  | Data collection | Hospital discharge data, survey |
|  | Weight calculation | Regression coefficients of Cox proportional hazard model |
| Lee, 2006  Country: United States  Sample size: 11,701  Mean age: 67  % women: 56  Follow-up: 1 year | Included diseases | Diabetes, cancer, lung disease, heart failure |
|  | Other variables | Difficulty with bathing, difficulty with finances, difficulty pulling and pushing objects, difficulty walking, smoking status, BMI, age, sex |
|  | Data collection | Patient self-report, administrative data |
|  | Weight calculation | Regression coefficients of logistic regression model |
| Fan, 2002  Country: United States  Sample size: 21,524  Mean age: 68  % women: 3  Follow-up: 2 years | Included diseases | Prior myocardial infarction, lung disease, pneumonia^a^, stroke, diabetes, cancer, congestive heart failure |
|  | Other variables | Age, sex, smoking status |
|  | Data collection | Patient self-report, administrative data |
|  | Weight calculation | Regression coefficients of Cox proportional hazard model |
| Quan, 2011  Countries: Australia, Canada, France, Japan, New Zealand, Switzerland  Sample size: 55,929  Mean age: not reported  % women: 65  Follow-up: 1 year | Included diseases | Congestive heart failure, renal disease, dementia, any malignancy (including leukaemia and lymphoma), chronic pulmonary disease, mild liver disease^a^, moderate or severe liver disease^a^, rheumatologic disease^a^, metastatic solid tumor, AIDS/HIV^a^, diabetes with chronic complications^b^, hemiplegia/paraplegia |
|  | Other variables | Age, sex |
|  | Data collection | Hospital discharge data, administrative data |
|  | Weight calculation | Regression coefficients of Cox proportional hazard model |
| Robusto, 2016  Country: Italy  Sample size: 999,391  Mean age: 60  % women: 54  Follow-up: 6 years | Included drugs | Antiarrythmics, drugs for acid related disorders, immunosuppressants, lipid modifying agents, platelet aggregation inhibitors, parenteral anticoagulants, systemic corticosteroids, oral anticoagulants, opioids, antineoplastics agents, anti-Parkinson drugs, inhaled bronchodilators, anti-psychotics, drugs for arterial hypertension, anti-dementia drugs, antihyperglycemic therapy, drugs for hypertensive heart disease |
|  | Other variables | Age, sex |
|  | Data collection | Hospital discharge data, prescription and administrative data |
|  | Weight calculation | Regression coefficients of Cox proportional hazard model |
| Von Korff, 1992  Country: United States  Sample size: 122,911  Mean age: not reported  % women: not reported  Follow-up: 1 year | Included drugs | Anti-coagulants, haemostatics, cardiac agents, diuretic loop, ACE inhibitors, insulin, oral hypoglecemics, anticonvulsants, cromolyn, epinephrine, isoproterenol, resp. products incl. bronchodilators and excl. cromolyn, beta-adrenergic, xanthine products, antiacne tretinoin, topical macrolides, cimetidine, ophthalmic miotics, uric acid agents, glucocorticoids, antilipemics, gold salts, ergot derivatives, antineoplastics, antitubercular agents, levodopa, antihypertensives, diuretics, beta blockers, calcium channel blockers |
|  | Other variables | Age, sex |
|  | Data collection | Prescription data, administrative data |
|  | Weight calculation | Expert opinion |

^a:^ Diseases which were not ascertained in the Rotterdam Study

^b^: Diseases which were ascertained in the Rotterdam Study using a broader category than the original index

**Appendix 1b: Characteristics of the indices**

The number of different predictors (diseases, drug classes or lifestyle data) included in one index ranged from 7 to 27. Among the five indices based on disease diagnoses, the number of included diseases varied between four (Lee, 2006) and twelve (Quan, 2011). The total number of different diseases used across all disease-based indices was 19. Cancer, diabetes, ischemic heart diseases and respiratory diseases were included in all five disease-based indices, either as one broad disease category or as a specific sub-type based on location, stage or severity. Four indices included stroke; two included renal failure and dementia; and one index included liver diseases, rheumatologic diseases, anaemia and HIV/AIDS. Both of the drug-based indices included drugs for treating ischemic heart diseases, cancer, diabetes, respiratory diseases, anti-Parkinson drugs, drugs for metabolic syndromes and drugs for gastrointestinal disorders. One of the two drug-based indices also included drugs for neurological and neurodegenerative diseases, dermatological diseases, rheumatic diseases, mental health disorders, and drugs for tuberculosis.

**Appendix 2: Ascertainment of diseases and lifestyle data collection**

*Cancer*

Diagnosis of incident cancer was based on medical records of general practitioners (including hospital discharge letters); through linkage with the national hospital discharge registry (Landelijke Medische Registratie); and with histology and cytopathology registries in the region (part of the nationwide network PALGA). Cancer diagnosis was coded independently by two physicians and classified according to the International Classification of Diseases, 10th revision (ICD‐10). In case of discrepancy between sources, consensus was sought through consultation with a physician specialised in internal medicine. (1)

As Desai (2002) used ICD-9 codes to identify different cancer types, we mapped their coding to ICD-10 as follows:

| **Cancer type** | **ICD-9** | **ICD-10** |
| --- | --- | --- |
| Solid tumor localised | 140 – 195.8 | C00 – C26, C30 – C34, C37 – C41, C43, C44, C46, C47, C50 – C57, C61 – C76 |
| Metastatic cancer | 196 – 199.0 | C77 – C80 |
| Lymphoma/leukemia | 200 – 208.91 | C81 – C85, C88, C90 – C95 |

Cancer subtypes for index by Quan (2011) were determined using the ICD-10 codes defined in the original article.

Lung cancer, colon cancer and cancer of the rectum necessary for two comparator models (count mortality and exact mortality) were identified using ICD-10 codes: C34 for Lung cancer, C18 for colon cancer and C20 for cancer of the rectum.

*Coronary heart disease and heart failure*

Clinical information on prevalent cardiovascular status was obtained using an automated follow-up system involving digital linkage of the study database to medical records by general practitioners in the research area. Trained research assistants collected notes, outpatient clinic records, hospital discharge letters, electrocardiograms, and imaging results from general practitioner records and hospital records. Furthermore, before the entry examination and every repeat examination at the Rotterdam Study research centre, participants were interviewed on the occurrence of cardiac events in the past (or since their last visit in case of repeat visit). Potential events were evaluated by research physicians and medical specialists.

The diagnosis of coronary heart disease includes the following outcomes: myocardial infarction, unrecognised myocardial infarction, coronary heart disease mortality and overall coronary heart disease. Diagnosis of myocardial infarction was determined by ECG measurements, which were evaluated using the Modular ECG Analysis System (MEANS) by two research physicians and a cardiologist. Diagnosis of heart failure was determined by using a validated score, based on the presence of at least two signs or symptoms suggestive of heart failure: shortness of breath, ankle swelling and pulmonary crepitations; or use of medication for the indication of heart failure, in combination with objective evidence of cardiovascular disease. (2, 3)

*Chronic kidney disease*

Chronic kidney disease was estimated by glomerular filtration rate (eGFR). eGFR was calculated according to the CKD Epidemiology Collaboration (CKD-EPI) equation of 2012, based on age, sex, race and serum creatinine. Serum creatinine was determined at baseline and during follow-up physical examinations using an enzymatic assay method, performed by the Erasmus MC AKC laboratory. Creatinine levels were calibrated by aligning its mean values with those of the Third National health and Nutrition Examination Survey (NHANES III) in different sex- and age-specific categories. Reduced kidney function, defined as a single assessment of eGFR <60 ml/min per 1·73 m^2^ at follow-up, was used as a proxy for incident chronic kidney disease.(4)

*Stroke and transient ischemic attack*

Stroke was defined according to the World Health Organization criteria. Information on stroke was collected continuously from medical records of general practitioners. Research physicians reviewed potential strokes using hospital discharge letters and information from general practitioners, and a consensus panel led by a consultant neurologist verified the stroke diagnoses. Major stroke was defined as stroke with severity greater than 3 on the NIH Stoke Scale. (5)

Transient ischemic attack (TIA) was defined as either focal or mixed transient neurological attack (TNA). TNA was defined as attacks of sudden neurological symptoms that completely resolved within 24 hours, with no clear evidence for the diagnosis of migraine, epilepsy, Meniere disease, hyperventilation, cardiac syncope, hypoglycemia, or orthostatic hypotension. If only focal brain symptoms (hemiparesis, hemihypesthesia, dysphasia, dysarthria, amaurosis fugax, hemianopia, hemiataxia, diplopia, or vertigo) were reported, the event was classified as a focal TNA. Non-focal-only brain symptoms (decreased consciousness, unconsciousness, confusion, amnesia, unsteadiness, non-rotatory dizziness, positive visual phenomena, cardiac or vegetative signs, paresthesias, bilateral weakness, and unwell feelings) were not classified as TIA. If both focal and non-focal symptoms were reported for the same attack, a mixed TNA was diagnosed**.** (6)

*Chronic obstructive pulmonary disease (COPD) and asthma*

COPD was diagnosed by an obstructive spirometry (FEV1/FVC < 70%) performed at the research centre visit. In absence of an interpretable study-acquired spirometry, the medical records were reviewed of all patients who regularly used medication for obstructive lung disease (Anatomical Therapeutic Chemical Classification codes: R03). Each such potential case was subsequently validated through careful evaluation of all medical records, hospitalisations and specialist letters and only included if a clear and well-founded diagnosis of COPD was retained. (7)

Asthma cases were defined as participants with a physician's diagnosis of asthma reported in their medical file. The identification of subjects with a diagnosis of asthma consisted of two steps: case finding and validation. The case finding phase bundled all participants with either: ever asthma and/or respiratory complaints in the questionnaire, the use of respiratory medication, an obstructive pulmonary function test, asthma as reported in the patient's medical file, hospital admissions for asthma, or a death record that reported asthma. Next, the medical files of all these possible asthma cases were systematically reviewed to search for asthma as diagnosed by a physician. Cases of asthma were defined as diagnosed by either a pulmonary physician/ asthma allergist or by a GP (as evidenced in the medical files). (8)

*Type 2 diabetes*

At baseline and during follow-up, cases of type 2 diabetes were ascertained by use of medical records from general practitioners (including laboratory glucose measurements), hospital discharge letters, and serum glucose measurements performed at regular physical examination at the Rotterdam Study research centre. Type 2 diabetes was defined as a fasting blood glucose concentration of 7·0 mmol/L or higher, a non-fasting blood glucose concentration of 11·1 mmol/L or higher (when fasting samples were unavailable), or the use of blood glucose-lowering drugs. Information about the use of blood glucose-lowering drugs was obtained from both structured home interviews and pharmacy dispensing records. (9)

*Dementia*

Participants were screened for dementia at baseline and subsequent centre visits with the Mini-Mental State Examination and the Geriatric Mental Schedule organic level. Those with a Mini-Mental State Examination score <26 or Geriatric Mental Schedule score >0 underwent further investigation and informant interview, including the Cambridge Examination for Mental Disorders of the Elderly. All participants also underwent routine cognitive assessment. In addition, the entire cohort was continuously under surveillance for dementia through electronic linkage of the study database with medical records from general practitioners and the regional institute for outpatient mental health care. A consensus panel led by a consultant neurologist established the final diagnosis according to standard criteria for dementia (DSM-IIIR), Alzheimer's disease (NINCDS–ADRDA) and vascular dementia (NINDS-AIREN). (10)

*Depression*

At baseline, all participants filled out either the Dutch versions of the Center for Epidemiologic Studies Depression Scale (CES-D) or the Dutch version of the Hospital Anxiety and Depression Scale. A score of 16 or higher on the CES-D was considered indicative of a depressive disorder; a score of 9 or higher on the Hospital Anxiety and Depression Scale was considered as the cutoff for depression. Cases of depression identified at baseline were considered prevalent. During the repeat examinations, all participants were screened with the CES-D as part of the home interview. The screen-positive participants were invited for a clinical interview conducted by a psychiatrist, psychogeriatrician, or clinical psychologist. With a computerized diagnostic algorithm based on the item scores, major and minor depressive disorders and dysthymia were classified according to DSM-IV-TR criteria. Active surveillance for the occurrence of depression took place during the follow-up, using a number of predefined cues such as symptoms of depression, prescriptions of psychiatric medication, the occurrence of major life events, and psychosocial problems. Two categories of depression were defined: depressive syndromes, including DSM-IV-TR depressive disorders and bipolar disorder, and clinically relevant depressive symptoms. The category of depressive syndromes consisted of major depressive disorder (MDD) and dysthymia together and other depressive syndromes. The group of other depressive syndromes covered the following: (1) depression recorded by a GP or physician; (2) self-reported depression for which the participant consulted a GP or a mental health professional; and (3) DSM-IV minor depression. The category of clinically relevant depressive symptoms included the following: (1) clinically relevant core symptom of major depression recorded during the psychiatric interview or in the medical record; (2) self-reported depression of a participant who did not consult a GP or a mental health professional; and (3) initiation of antidepressant drug treatment (without documentation of clinical symptoms). Grief, adjustment disorder, and burnout, characterized by emotional exhaustion and reduced satisfaction in personal accomplishment were not regarded as depression. All information that indicated potential depression was reviewed by two physicians and a research psychologist, a final diagnosis was determined at consensus meetings. Both depression categories were included in the count model. The count mortality model and the exact mortality model only included major depressive disorder and dysthymia; patients with depressive syndromes and clinically relevant depressive symptoms were considered free of depression.

*Lifestyle data*

Smoking status was obtained through interviews with participants conducted upon entering the Rotterdam Study and then repeated every three to five years. Similarly, activities of daily living (difficulty bathing, difficulty with finances, difficulty getting up from bed, difficulty walking on a flat terrain) were assessed using the Stanford Health Assessment Questionnaire and the Instrumental Activities of Daily Living scale. BMI and hemoglobin levels were collected during physical examination at the Rotterdam Study Research Centre conducted at the same time as the repeat interviews.

| **Appendix 3a \| Disease mapping from the original disease definition onto data available within Rotterdam Study.** | | | |
| --- | --- | --- | --- |
| **Index** | **Original disease definition** | Weight | **Mapped onto Rotterdam Study disease definitions** |
| Tooth, 2008  Disease status ascertained by self-report | Heart disease | 1 | Coronary heart disease + heart failure |
|  | Stroke | 2 | Stroke |
|  | Low iron | 1 | Hemoglobin levels:   - < 8·1 mmol/L (13 g/dL) for men - <7·5 mmol/L (12 g/dL) for women |
|  | Bronchitis/emphysema | 2 | COPD + asthma |
|  | Diabetes | 1 | Diabetes |
|  | Cancer | 3 | Cancer (all types excluding non-melanoma skin cancer) |
|  | Alzheimer's disease | 4 | Dementia |
| Desai, 2002  Disease status ascertained by hospital discharge diagnoses (ICD-9 codes) | CHF/cardiomyopathy | 2 | Heart failure |
|  | Pneumonia | 1 | MISSING |
|  | COPD/chronic lung disease | 2 | COPD + asthma |
|  | Cancer (solid tumor, localized) | 3 | Cancer (solid tumor, localised) |
|  | Cancer (metastatic) | 3 | Cancer (metastatic) |
|  | Lymphoma/leukemia | 6 | Lymphoma/leukemia |
|  | Major stroke (hemiplegia) | 2 | Stroke with NIH Stroke Scale ≥ 3 |
|  | Acute renal failure | 5 | MISSING |
|  | Chronic renal failure | 2 | Chronic kidney disease |
|  | Diabetes mellitus with end-organ damage | 1 | Diabetes |
| Lee, 2006  Disease status and other variables ascertained by self-report | Diabetes | 1 | Diabetes |
|  | Cancer | 2 | Cancer (all types excluding non-melanoma skin cancer) |
|  | Lung disease | 2 | COPD + asthma |
|  | Heart failure | 2 | Heart failure |
|  | BMI > 25 | 1 | BMI |
|  | Smoking | 2 | Current smoker |
|  | Difficulty with bathing | 2 | Difficulty bathing |
|  | Difficulty with finances | 2 | Difficulty with finances |
|  | Difficulty pulling and pushing | 1 | Difficulty getting up from bed |
|  | Difficulty walking | 2 | Difficulty walking on a flat terrain |
|  | Age below 65 | 1 |  |
|  | Age 65-69 | 2 |  |
|  | Age 70-74 | 3 |  |
|  | Age 75-79 | 4 |  |
|  | Age 80-84 | 5 |  |
|  | Age 85 and more | 7 |  |
|  | Male sex | 2 |  |
| Fan, 2002  Disease status ascertained by self-report | Prior myocardial infarction | 1 | Myocardial infarction |
|  | Lung disease | 1 | COPD + asthma |
|  | Pneumonia | 1 | MISSING |
|  | Stroke | 2 | Stroke |
|  | Diabetes | 2 | Diabetes |
|  | Cancer | 2 | Cancer (all types excluding non-melanoma skin cancer) |
|  | Congestive heart failure | 2 | Heart failure |
|  | Curent smoker | 4 | Current smoker |
|  | Past smoker | 2 | Ever smoker |
|  | Age 55-59 | 1 |  |
|  | Age 60-64 | 2 |  |
|  | Age 65-69 | 3 |  |
|  | Age 70-74 | 4 |  |
|  | Age 75-79 | 5 |  |
|  | Age 80-84 | 6 |  |
|  | Age 85-89 | 7 |  |
|  | Age 90-94 | 8 |  |
|  | Age 95-99 | 9 |  |
| Quan, 2011  Disease status ascertained by hospital discharge diagnoses (ICD-10 codes) | Congestive heart failure | 1 | Heart failure |
|  | Dementia | 2 | Dementia |
|  | Chronic pulmonary disease | 1 | COPD |
|  | Rheumatologic disease | 1 | MISSING |
|  | Mild liver disease | 2 | MISSING |
|  | Diabetes with chronic complications | 1 | Diabetes |
|  | Hemiplegia or paraplegia | 2 | Stroke with NIH Stroke Scale ≥ 3 |
|  | Renal disease | 1 | Chronic kidney disease |
|  | Any malignancy, including leukemia and lymphoma | 2 | Cancer excluding metastatic solid tumor and non-melanoma skin cancer |
|  | Moderate or severe liver disease | 4 | MISSING |
|  | Metastatic solid tumor | 6 | Metastatic solid tumor |
|  | AIDS/HIV | 4 | MISSING |

COPD: Chronic Obstructive Pulmonary Disease

BMI: Body Mass Index

| **Appendix 3b \| Weghts assigned to different drug classes and their corresponding ATC codes** | | | |
| --- | --- | --- | --- |
| **Index** | **Original disease definition** | Weight | **ATC codes** |
| Robusto 2016 | Antiarrythmics | 1 | C01B |
|  | Immunosuppressants | -1 | L04 |
|  | Platelet aggregation inhibitors | 2 | B01AC |
|  | Parenteral anticoagulants | 1 | B01AB or B01AX |
|  | Oral anticoagulants, | 1 | B01AA |
|  | Antineoplastics agents | 3 | L01s |
|  | Inhaled bronchodilators | 2 | R03A, R03BB or R03DA |
|  | Drugs for arterial hypertension, | 1 | C02A, C02C, C02LA, C02LB, C03A, C03BA, C03EA01, C07AA, C07AG, C07BB, C07C, C08, C09AA, C09BA, C09CA, C09DA, |
|  | Antihyperglycemic therapy, | 2 | A10 |
|  | Drugs for hypertensive heart disease | 3 | C01AA05, C03CA01, C03DA01, C07Ag02, C07AB07, C07AB03,C09, C01AA05 |
|  | Drugs for acid related disorders | 1 | A02 |
|  | Lipid modifying agents | -2 | C10 |
|  | Systemic corticosteroids | 2 | H02AB |
|  | Opioids | 6 | N02A (other than codein and tramadol) |
|  | Anti-Parkinson rugs | 4 | N04 |
|  | Antipsychotics | 3 | N05A |
|  | Anti-dementia drugs | 4 | N06A |
| Von Korff, 1992 | Anti-coagulants, hemostatics | One class: 3  Two classes: 4  Three classes: 5 | B01A, B02 |
|  | Cardiac agents, ACE inhibitors |  | C01, C09A, C09B |
|  | Diuretic loop |  | C03C |
|  | Epinephrine, isoproterenol | One class: 2  Two or more classes: 3 | C01CA |
|  | Resp. products incl bronchodilators excl cromolyn. |  | R05CB, R03BA, R03BB, R01, R02, R06, R07 |
|  | Beta-adrenergic, misc. |  | R03C, R03A |
|  | Xanthine products |  | R03DA |
|  | Glucocorticoids | 3 | H02AB |
|  | Gold salts | 3 | M01CB |
|  | Antineoplastics | 3 | L01 |
|  | Levodopa | 3 | N04BA |
|  | Antihypertensives (except ACE inhibitors), Calcium channel blockers | 2 | C02, C08 |
|  | Diuretics, Beta blockers | 1 (if taken without antihypertensives) | C03A, C03B, C03D, C03E, C03X, C07 |
|  | Insulin | 2 | A10A |
|  | Oral hypoglecemics | 2 | A10B |
|  | Anticonvulsants | 2 | N03 |
|  | Cromolyn | 2 | R03BC01 |
|  | Antiacne tretinoin | 1 | D10AD01 |
|  | Topical macrolides | 1 | D10AF |
|  | Cimetidine | 1 | A02BA01 |
|  | Ophthalmic miotics | 1 | S01E |
|  | Uric acid agents | 1 | M04AA, M04AB |
|  | Antilipemics | 1 | C10 |
|  | Ergot derivatives | 1 | N02CA |
|  | Antitubercular agents | 1 | J04 |

| **Appendix 4. Performance evaluation methods** | | | | |
| --- | --- | --- | --- | --- |
| **Method** | **Purpose** | **Description** | **Interpretation/range** | **Reported as** |
| C-statistic | To measure how well a model discriminates between individuals with high and low risk of mortality on a relative scale. | Probability that for a randomly selected pair of observations – one event and one non-event - the model assigns higher probability to the observation with event. | From 0 to 1. Values above 0.5 mean improvement with respect to a random guess, 1 means perfect discrimination. | Forest plot (Figure 1) and in absolute value (Table 2). |
| Integrated Discrimination Index (IDI) | To measure the improvement in discrimination between individuals with high and low risk on the scale of predicted probabilities. | Difference in mean predicted probabilities between observations with and without events with respect to a reference model. | Positive values indicate improved discrimination, zero indicates no change. Exact range of values depends on the discrimination of the reference model. | IDI with “base model” as the reference model. |
| Brier Score (18) | To measure the accuracy of the model predictions. | Mean squared error of the predictions (i.e. mean distance between the actual binary outcomes and its predicted probabilities) | From 0 to 1, actual values depend on overall mortality risk in the sample population. Lower scores indicate higher accuracy. | The Brier Skill Score, which measures *percent* change in Brier Score with respect to the “base model’. |
| Net Reclassification Improvement (NRI) | To measure the improvement in probabilities separately for observations with and without event with respect to a reference model | Proportion of observations whose probabilities moved in the right direction (i.e. Increased for events and decreased for non-events) minus the proportion whose probabilities move in the opposite direction. | From –1 to 1. Negative values mean poorer performance than the reference model, positive values mean improved performance. | Separately NRI for events and non-events, with the "base model” as the reference model. |
| Calibration | To measure how well the assigned probabilities match the actual observed outcomes. | The proportion of observations with the same or similar assigned probabilities which experienced the event. | In calibration plot: straight line with slope 1 indicates perfect calibration (i.e. predicted probabilities perfectly match actual outcomes) | Calibration plots (Figure 1) |

**Appendix 5: Formulas for the performance metrics**

| **C-statistics (c)**  :$c = \frac{C}{C+D}$  C: number of concordant pairs of observations (i.e. Estimated probability is higher for the observation that experienced the event),  D: number of discordant pairs of observations (i.e. Estimated probability is higher for the observation that experienced the event)  C+D is therefore the total number of possible pairs of observations in which one observation of the pair did experience the event and the other did not. |
| --- |
| **Integrated Discrimination Index (IDI)**  𝐼𝐷𝐼= (𝑝_𝑛𝑒𝑤, 𝑐𝑎𝑠𝑒s_−𝑝_𝑟𝑒𝑓, 𝑐𝑎𝑠𝑒𝑠_)−(𝑃_𝑒ne𝑤,𝑛𝑜𝑛𝑐𝑎𝑠𝑒𝑠_−𝑝_𝑟𝑒𝑓, 𝑛𝑜𝑛𝑐𝑎𝑠𝑒𝑠_)  *p*: average estimated probabilities,  new: probabilities of the model being assessed  ref: probabilities of the reference model  cases: observations that experienced the event  noncases: observations that did not experienced the event)  (ie. 𝑝_𝑛𝑒𝑤, 𝑐𝑎𝑠𝑒s_ is the average probability for observations which experienced the event as estimated by the model being assessed) |
| **Brier Score (BS)**  $BS = \frac{1}{N}\sum_{t = 1}^{N} \left( f_{t}-o_{t} \right)^{2}$  f_t_ estimated probability of event  o_t_ actual outcome (0 or 1) |
| **Net Reclassification Improvement (NRI)**  𝑁𝑅𝐼𝑒vent = 𝑝(𝑢𝑝 \| 𝑒𝑣𝑒𝑛𝑡) − 𝑝(𝑑𝑜𝑤𝑛 \| 𝑒𝑣𝑒𝑛𝑡)  𝑁𝑅𝐼𝑛𝑜𝑛𝑒𝑣𝑒𝑛𝑡 = 𝑝(𝑢𝑝 \| 𝑛𝑜𝑛𝑒𝑣𝑒𝑛𝑡) − 𝑝(𝑑𝑜𝑤𝑛 \| 𝑛𝑜𝑛𝑒𝑣𝑒𝑛𝑡)  p: proportion of observations reclassified up or down and separately calculated for observations with and without the event (ie. P( up \| event) is the proportion of observations which experienced the event reclassified up as compared to the reference model) |

**Appendix 6: Results of the sensitivity analyses**

Three indices were developed for populations of a specific age (70 years and older: Tooth, 2008; Desai, 2002) or of a specific sex (Tooth, 2008; Fan, 2002). To assess generalisability to other groups, we applied these indices to a sample without any age or sex restrictions and compared them to the count and base models fitted to the same data.

Basic characteristics of the new samples are in **A4 Table 1**. **A4 Table 2** below shows the performance statistics for the three models (index, count and base) applied to two samples: the original sample with age restriction and a new sample without any restriction.

Two indices (Desai, 2002; Quan, 2011) only considered diabetes with end-organ damage or diabetes with chronic complications. As data on diabetes severity were not available within the Rotterdam Study, we calculated the upper bound of the indices performance by considering the hypothetical scenario in which diabetes with end-organ damage or chronic complications always led to mortality. Participants with diabetes who died during follow-up kept their diagnoses of diabetes, while participants with diabetes who did not die were considered as having diabetes without end-organ damage or chronic complications.

In this case, the sample population was the same but the index score changed: **A4 Table 3** shows the performance statistics for the two indices, compared to the original version with full, unaltered data on diabetes prevalence (i.e. including those with diabetes without end-organ damage).

| A6 Table 1 \| Baseline characteristics of the sample populations with unrestricted age and/or sex | | | |
| --- | --- | --- | --- |
|  | **Tooth, 2008 (N=8,863)** | **Desai, 2002 (N=9,044)** | **Fan, 2002 (N=7,068)** |
| Mean age, years (sd) | 64.7 (9.6) | 64.7 (9.6) | 67.4 (10.3) |
| Women (%) | 5,012 (56.5) | 5,164 (57.0) | 4,125 (58.3) |
| Died during follow-up | 821 (9.0) | 83 (1.0) | 206 (3.0) |

| **A6 Table 2 \| Predictive performance for unrestricted age and/or sex (compared to samples with restricted age and/or sex).** | | | | | | |
| --- | --- | --- | --- | --- | --- | --- |
| **Sample for** | **Metric** | **Base** | **Index** | **Count** | **Count mortality** | **Exact mortality** |
| **Tooth, 2008**  **Restricted age and sex (original)** | C-statistic | 0.72 (0.69:0.75) | 0.75 (0.73:0.78) | 0.74 (0.72:0.77) | 0.73 (0.70:0.76) | 0.73 (0.71:0.77) |
|  | BSS | 0.100 (0.09:0.108) | 0.03 (0.01:0.05) | 0.02 (0.01:0.04) | 0.01 (0:0.02) | 0.02 (0.01:0.05) |
|  | NRI - events | . | -0.06 (-0.14:0.02) | 0.23 (0.12:0.34) | -0.01 (-0.12:0.1) | -0.35 (-0.55:-0.03) |
|  | NRI - non-events | *.* | 0.42 (0.36:0.48) | 0.19 (0.14:0.23) | 0.47 (0.44:0.51) | 0.70 (0.51:0.82) |
|  | IDI | *.* | 0.03 (0.01:0.05) | 0.02 (0.01:0.04) | 0.01 (0:0.02) | 0.03 (0.01:0.05) |
| **Tooth, 2008**  **Unrestricted age and sex** | C-statistic | 0.79 (0.78:0.80) | 0.81 (0.80:0.83) | 0.81 (0.80:0.83) | 0.81 (0.79:0.82) | 0.80 (0.78:0.82) |
|  | BSS | 0.073 (0.068:0.079) | 0.03 (0.02:0.04) | 0.03 (0.02:0.04) | 0.02 (0.01:0.03) | 0.01 (0.00:0.03) |
|  | NRI - events | *.* | -0.06 (-0.12:-0.00) | 0.06 (0.01:0.11) | 0.05 (-0.01:0. 11) | 0.05 (-0.03:0.16) |
|  | NRI - non-events | *.* | 0.46 (0.44:0.49) | 0.31 (0.29:0.34) | 0.40 (0.32:0.46) | -0.19 (-0.28:-0.08) |
|  | IDI | . | 0.03 (0.02:0.04) | 0.03 (0.02:0.04) | 0.02 (0.01:0.03) | 0.02 (-0.01:0.03) |
| **Fan,** **2002**  **Restricted sex (original)** | C-statistic | 0.76 (0.7:0.82) | 0.80 (0.75:0.85) | 0.81 (0.76:0.86) | 0.79 (0.74:0.84) | 0.79 (0.76:0.85) |
|  | BSS | 0.033 (0.026:0.04) | 0.03 (0.00:0.08) | 0.04 (0.01:0.10) | 0.02 (0.00:0.06) | 0.00 (0.00:0.09) |
|  | NRI - events | *.* | 0.17 (-0.03:0.38) | 0.18 (-0.03:0.41) | 0.14 (-0.04:0.31) | 0.24 (-0.1:0.51) |
|  | NRI - non-events | *.* | 0.22 (0.00:0.34) | 0.51 (0.32:0.63) | 0.31 (0.24:0.41) | 0.51 (0.00:0.68) |
|  | IDI | . | 0.03 (0.00:0.08) | 0.05 (0.02:0.09) | 0.02 (0.01:0.05) | 0.05 (0.02:0.10) |
| **Fan,** **2002**  **Unrestricted sex** | C-statistic | 0.78 (0.74:0.81) | 0.79 (0.76:0.83) | 0.81 (0.77:0.85) | 0.80 (0.77:0.84) | 0.80 (0.77:0.84) |
|  | BSS | 0.028 (0.024:0.032) | 0.02 (-0.01:0.05) | 0.04 (0.02:0.07) | 0.02 (0.01:0.04) | 0.01 (0.00:0.04) |
|  | NRI - events | *.* | 0.14 (-0.03:0.28) | 0.27 (0.14:0.40) | 0.12 (0.01:0.24) | 0.16 (0.00:0.36) |
|  | NRI - non-events | *.* | 0.13 (-0.03:0.28) | 0.40 (0.31:0.47) | 0.34 (0.29:0.40) | 0.28 (-0.04:0.46) |
|  | IDI | . | 0.02 (0.00:0.04) | 0.04 (0.02:0.06) | 0.02 (0.01:0.04) | 0.03 (0.01:0.05) |
| **Desai, 2002**  **Restricted age**  **(original)** | C-statistic | 0.76 (0.71:0.81) | 0.82 (0.78:0.87) | 0.80 (0.75:0.85) | 0.81 (0.77:0.86) | 0.82 (0.79:0.87) |
|  | BSS | 0.029 (0.023:0.035) | 0.01 (0.00:0.05) | 0.02 (0.01:0.06) | 0.03 (0.01:0.07) | 0.02 (0.01:0.09) |
|  | NRI - events | *.* | 0.15 (-0.01:0.32) | 0.15 (-0.05:0.36) | 0.23 (0.05:0.41) | 0.38 (0.12:0.65) |
|  | NRI - non-events | *.* | 0.41 (0.30:0.50) | 0.47 (0.34:0.51) | 0.44 (0.30:0.60) | 0.32 (0.25:0.46) |
|  | IDI | . | 0.03 (0.01:0.05) | 0.03 (0.01:0.06) | 0.04 (0.02:0.06) | 0.06 (0.03:0.10) |
| **Desai, 2002**  **Unrestricted age** | C-statistic | 0.80 (0.75:0.85) | 0.82 (0.78:0.86) | 0.82 (0.79:0.86) | 0.82 (0.78:0.86) | 0.82 (0.78:0.87) |
|  | BSS | 0.009 (0.007:0.011) | 0.00 (0.00:0.02) | 0.02 (0.00:0.04) | 0.01 (0.00:0.04) | 0.01 (-0.01:0.05) |
|  | NRI - events | *.* | 0.06 (-0.10:-0.22) | 0.05 (-0.11:0.20) | 0.11 (-0.09:0.28) | -0.04 (-0.27:0.18) |
|  | NRI - non-events | *.* | 0.34 (0.24:0.42) | 0.32 (0.24:0.38) | 0.37 (0.24:0.46) | 0.12 (-0.20:-0.71) |
|  | IDI | . | 0.01 (0.00:0.02) | 0.02 (0.00:0.04) | 0.01 (0.00:0.03) | 0.02 (0.00:0.05) |

###

| **A6 Table 3 \| Predictive performance in a hypothetical scenario in which diabetes with end-organ damage always leads to mortality.** | | | | | | |
| --- | --- | --- | --- | --- | --- | --- |
| **Sample for** | **Metric** | **Base** | **Index** | **Count** | **Count mortality** | **Exact mortality** |
| **Quan, 2011**  **Original data** | C-statistic | 0.85 (0.81:0.88) | 0.89 (0.87:0.92) | 0.88 (0.85:0.90) | 0.88 (0.86:0.91) | 0.88 (0.86:0.92) |
|  | BSS | 0.014 (0.011:0.016) | 0.02 (0.00:0.04) | 0.02 (0.00:0.04) | 0.01 (0.00:0.03) | 0.01 (0:0.06) |
|  | NRI - events | *.* | 0.23 (0.08:0.36) | 0.24 (0.06:0.4) | 0.24 (0.08:0.4) | 0.31 (0.09:0.48) |
|  | NRI - non-events | *.* | 0.46 (0.36:0.53) | 0.39 (0.28:0.56) | 0.46 (0.39:0.51) | 0.27 (-0.01:0.49) |
|  | IDI | *.* | 0.03 (0.01:0.05) | 0.02 (0.01:0.04) | 0.02 (0.01:0.04) | 0.04 (0.02:0.07) |
| **Quan 2011**  **Altered diabetes data** | C-statistic | N/A | 0.91 (0.88:0.93) | N/A | N/A | N/A |
|  | BSS | N/A | 0.03 (0.01:0.07) | N/A | N/A | N/A |
|  | NRI - events | *.* | 0.28 (0.14:-0.41) | N/A | N/A | N/A |
|  | NRI - non-events | *.* | 0.55 (0.43:0.61) | N/A | N/A | N/A |
|  | IDI | . | 0.05 (0.03:0.08) | N/A | N/A | N/A |
| **Desai, 2002**  **Original data** | C-statistic | 0.76 (0.71:0.81) | 0.82 (0.78:0.87) | 0.80 (0.75:0.85) | 0.81 (0.77:0.86) | 0.82 (0.79:0.87) |
|  | BSS | 0.029 (0.023:0.035) | 0.01 (0.00:0.05) | 0.02 (0.01:0.06) | 0.03 (0.01:0.07) | 0.02 (0.01:0.09) |
|  | NRI - events | *.* | 0.15 (-0.01:0.32) | 0.15 (-0.05:0.36) | 0.23 (0.05:0.41) | 0.38 (0.12:0.65) |
|  | NRI - non-events | *.* | 0.41 (0.30:0.50) | 0.47 (0.34:0.51) | 0.44 (0.30:0.60) | 0.32 (0.25:0.46) |
|  | IDI | . | 0.03 (0.01:0.05) | 0.03 (0.01:0.06) | 0.04 (0.02:0.06) | 0.06 (0.03:0.10) |
| **Desai, 2002**  **Altered diabetes data** | C-statistic | N/A | 0.83 (0.79:0.87) | N/A | N/A | N/A |
|  | BSS | N/A | 0.02 (0.00:0.06) | N/A | N/A | N/A |
|  | NRI - events | *.* | 0.20 (-0. 04:-0.37) | N/A | N/A | N/A |
|  | NRI - non-events | *.* | 0.47 (0.35:0.55) | N/A | N/A | N/A |
|  | IDI | . | 0.03 (0.01:0.07) | N/A | N/A | N/A |

### **References**

1. van der Willik KD, Ruiter R, van Rooij FJA, Verkroost-van Heemst J, Hogewoning SJ, Timmermans K, et al. Ascertainment of cancer in longitudinal research: The concordance between the Rotterdam Study and the Netherlands Cancer Registry. Int J Cancer. 2020;147(3):633-40.

2. Bleumink GS, Knetsch AM, Sturkenboom MC, Straus SM, Hofman A, Deckers JW, et al. Quantifying the heart failure epidemic: prevalence, incidence rate, lifetime risk and prognosis of heart failure The Rotterdam Study. Eur Heart J. 2004;25(18):1614-9.

3. Leening MJ, Kavousi M, Heeringa J, van Rooij FJ, Verkroost-van Heemst J, Deckers JW, et al. Methods of data collection and definitions of cardiac outcomes in the Rotterdam Study. Eur J Epidemiol. 2012;27(3):173-85.

4. van Westing AC, Ochoa-Rosales C, van der Burgh AC, Chaker L, Geleijnse JM, Hoorn EJ, Voortman T. Association of habitual coffee consumption and kidney function: A prospective analysis in the Rotterdam Study. Clin Nutr. 2023;42(2):83-92.

5. Berghout BP, Bos D, Koudstaal PJ, Ikram MA, Ikram MK. Risk of recurrent stroke in Rotterdam between 1990 and 2020: a population-based cohort study. Lancet Reg Health Eur. 2023;30:100651.

6. Fani L, Bos D, Mutlu U, Portegies MLP, Zonneveld HI, Koudstaal PJ, et al. Global Brain Perfusion and the Risk of Transient Ischemic Attack and Ischemic Stroke: The Rotterdam Study. J Am Heart Assoc. 2019;8(7):e011565.

7. Lahousse L, Niemeijer MN, van den Berg ME, Rijnbeek PR, Joos GF, Hofman A, et al. Chronic obstructive pulmonary disease and sudden cardiac death: the Rotterdam study. Eur Heart J. 2015;36(27):1754-61.

8. de Roos EW, Lahousse L, Verhamme KMC, Braunstahl GJ, Ikram MA, In 't Veen J, et al. Asthma and its comorbidities in middle-aged and older adults; the Rotterdam Study. Respir Med. 2018;139:6-12.

9. Ligthart S, van Herpt TT, Leening MJ, Kavousi M, Hofman A, Stricker BH, et al. Lifetime risk of developing impaired glucose metabolism and eventual progression from prediabetes to type 2 diabetes: a prospective cohort study. Lancet Diabetes Endocrinol. 2016;4(1):44-51.

10. de Bruijn RF, Bos MJ, Portegies ML, Hofman A, Franco OH, Koudstaal PJ, Ikram MA. The potential for prevention of dementia across two decades: the prospective, population-based Rotterdam Study. BMC Med. 2015;13:132.
